# BlyS is up-regulated by hypoxia and promotes migration of human breast cancer cells

**DOI:** 10.1186/1756-9966-31-31

**Published:** 2012-03-31

**Authors:** Jing Zhu, Li Sun, Sensen Lin, Renping Zhao, Liqiang Zhou, Dongdong Fang, Liang Chen, Jin Liu, Wenting Shi, Luyong Zhang, Shengtao Yuan

**Affiliations:** 1 National Nanjing New Drug Screening Center, China Pharmaceutical University, Nanjing 210009, Peoples Republic of China; 2 Jiangsu Center for Pharmacodynamics Research and Evaluation, China Pharmaceutical University, Nanjing 210009, Peoples Republic of China

**Keywords:** Hypoxia, BLyS, Cell migration

## Abstract

**Background:**

The role of B Lymphocyte Stimulator (BLyS) in the survival of malignant B cells and the maintenance of normal B cell development and homeostasis has been intensively studied in the literature. However, the influence of BLyS on breast cancer progression remains unclear. The study aimed to investigate the effect of hypoxia on BLyS regulation, cell migratory response to BLyS and the possible molecular mechanisms.

**Methods:**

In this study, we examined the role of BLyS in the migration of human breast cancer cells by transwell assay. We also explored whether BLyS and its receptors expressed in human breast cancer cell lines by immunofluorescence and Western Blotting. Then we detected the expression level of BLyS in both normoxic and hypoxic conditions by real time-PCR and Western Blotting. Pathways involved were confirmed by Western Blotting, immunofluorescence, transwell assay and luciferase assay.

**Results:**

According to our study, the expression level of BlyS was increased in human breast cancer cell lines in hypoxic conditions. Up-regulation of this protein led to activation and nuclear translocation of NF-kappa B p65. We also found that the number of migrated cells was increased in the presence of BLyS and inhibition of phosphorylation of Akt attenuated the enhanced migratory response.

**Conclusions:**

It suggested that better understanding of BLyS, an immunopotentiator, may offer a potential therapeutic target for the treatment of human breast cancers. In addition, BLyS promoted breast cancer cells migration, underscoring the necessity of appropriate applications of immunopotentiators to cancer treatment.

## Background

B Lymphocyte Stimulator (BLyS), a key member of the tumor necrosis factor superfamily, binds to three receptors: B-cell maturation antigen (BCMA), transmembrane activator and CAML interactor (TACI), and B cell-activating factor receptor (BAFF-R). BLyS promotes survival of splenic immature transitional and mature B cells [1]. Over-expression of BLyS has been associated with multiple myeloma (MM) [2], Systemic lupus erythematosus (SLE) [3] and B cell lymphoma [4]. It has also been reported that this ligand/receptor dyad plays a critical role in the growth and survival of malignant plasma cells and B cells [5]. Recent studies in ductal breast cancer patients have suggested a role of BLyS in the development of breast cancer. But its molecular mechanisms remain to be elucidated [6].

Hypoxia plays a significant role in the pathogenesis of heart disease, cancer, neuron death, etc. [7]. Inflammatory factors have been shown to be transcriptional regulated by hypoxia induced factor-1α (HIF-1α) or NF-kappa B in hypoxic conditions [8]. The expression of BLyS is up-regulated by hypoxia, while the mechanism is still uncertain. We hypothesized that HIF-1α or NF-kappa B pathway might be responsible for the up-regulation. In addition, the inflammatory factors such as TNF-α, IL-1α lead to increased cancer cell migration [9]. Therefore, the human breast cancer cell migration in response to BLyS and possible molecular mechanisms were explored in this study.

## Methods

### Cell line and cell culture

Breast cancer cell lines MDA-MB-435, MDA-MB-231 and MDA-MB-468 and B cell line Ramos were purchased from Cell Bank of Institute of Biochemistry and Cell Biology, Chinese Academy of Sciences (Shanghai, China). MDA-MB-435 cells and Ramos cells were cultured in Dulbecco's Modified Eagle's Medium (Gibco, Grand Island, NY) and MDA-MB-231 cells and MDA-MB-468 cells were cultured in L-15 (Gibco, Grand Island, NY), containing 10% fetal bovine serum (Gibco, Grand Island, NY). The cells were used from three to six passages.

### Materials

Anti-human BLyS and anti-human TACI antibodies were obtained from R&D Systems (Minneapolis, MN). Anti-human BAFF-R and anti-human BCMA antibodies were purchased from Abcam Inc (Cambridge, MA). Anti-Lamin B, anti-NF-kappa B p65 antibodies and donkey anti-goat secondary antibodies were obtained from Santa-Cruz (Santa Cruz, CA). Anti-Akt, anti-p-Akt (Ser 473), anti-p38 MAPK, anti-p-p38 MAPK (Tyr 182), anti-HIF-1α antibodies and goat anti-rabbit secondary antibodies were obtained from Cell Signaling (Beverly, MA) Anti-β-actin antibody was obtained from Sigma (St. Louis, MO). Goat anti-mouse peroxidase-conjugated antibody was from Sigma (St. Louis, MO). RevertAid™ first strand cDNA Synthesis Kit, TurboFect™ in vitro transfection reagent and restriction enzymes Kpn I and Xho I were purchased from Fermentas (Shenzhen, China), Dual-luciferase assay system, pGL3-basic (promoterless) luciferase vector and pRL-SV40 plasmid were obtained from Promega (San Francisco, California, USA). API-1, SB 202190, PX 12 and Caffeic acid phenethyl ester (CAPE) were from Tocris (Bristol, UK). Recombinant human BAFF was purchased from R&D system (Minneapolis, MN). SYBR Premix Ex Taq II and pMD^® ^18-T Vector were purchased from TAKARA (Dalian, China). DNA purification kit, QIAprep spin miniprep kit and QIAquick gel extraction kit were purchased from Qiagen (Shanghai, China).

### Migration assay

Cell migration assay were performed in a double chamber transwell (Corning) with polycarbonate membranes (8.0 μm pore size). 8 × 10^4 ^cells were added to the upper chamber, treated with or without specific antagonists. Different concentrations of BLyS were added to the lower chamber. 1% FBS was used as a negative control. After incubation at 37 for 8 h in hypoxic or normoxic conditions, migrated cells were stained and counted in five randomly selected fields.

### Quantitative real-time PCR

Total RNA was extracted using a Trizol reagent (Invitrogen Corporation, Grand Island, NY, USA) and was reversed to cDNA using RevertAid™ first strand cDNA Synthesis Kit according to the manufacturer's instructions. All primers were synthesized by Sangon Biotech (Shanghai, China) or TAKARA (Dalian, China). The primers used in Q-PCR are listed as follow: BLyS (GenBank, NM_006573.4) 5'- CGT GCC GTT CAG GGT CCA G-3' (forward) and 5'-TCG AAA CAA AGT CAC CAG ACT CAA T-3' (reverse); β-actin (GenBank, AF035119) 5'-CTC CTC CTG AGC GCA AGT ACT C-3' (forward) and 5'-CGG ACT CGT CAT ACT CCT GCT-3' (reverse). The gene levels in the resultant cDNAs were examined using the detection system (TAKARA) with SYBR-green as fluorescent dye enabling real time detection of PCR products according to the manufacturer's protocol. The relative expression levels of the genes were determined against β-actin levels in the samples.

### Western blotting analysis

Total cell lysates were prepared in RIPA buffer supplemented with protease inhibitors. The proteins were fractionated by 8%-12% sodium dodecyl sulfate-polyacrylamide gel electrophoresis (SDS-PAGE) and electroblotted onto nitrocellulose membrane (Bio-Rad). The membranes were probed with primary antibodies and then probed with relative secondary antibody. β-actin was used as a loading control.

### Immunofluorescence

For BLyS and its three receptors staining in cells, MDA-MB-435, MDA-MB-231, MDA-MB-468 cells and Ramos cells were seeded on coverslips and cultured in 5% CO_2 _incubator. At 12 h after subculture, the plate with Ramos cells was centrifuged at 1, 000 rpm for 10 min and all the cells were fixed in 4% paraformadehyde for 10 min, washed and incubated with anti-BLyS antibody, anti-BAFF-R antibody, anti-BCMA antibody and anti-TACI antibody (1:100 dilution in 1% BSA-PBS). The cells were then incubated with relative FITC-conjugated secondary antibody (1:1000 dilution in 1% BSA-PBS) for 1 h at room temperature and with Hoechst 33342 for 30 minutes. The processed cells were mounted and fluorescence microscopy images were taken from five random fields in each slide using an inverted microscope (Olympus IX 71, Japan).

### Plasmid construction, transient transfection and luciferase assays

pGL3-Basic luciferase vector, a plasmid of luciferase-reporter for human BLyS promoter (GenBank, NT_009952.14, -1082 to +118), was used to prepare the reporter constructs. DNA was extracted from MDA-MB-435 cells. BLyS promoter was amplified by PCR using following primers: 5'- GCG GTA CCA AGC CTG GGT CTG GAG TTC T-3' (forward) and 5'- GCC TCG AGC CTT TCT GCC TTT CTG CAT C-3' (reserve). Cloned fragments were recovered and ligated into pGL3-basic luciferase vector. DNA transfectants were prepared using QIAprep spin miniprep kit. Cells were cultured in 24-well plates to 70-80% of confluence, and then transfected with 1 μg of pGL3-Basic/BP or pGL3-Basic. Plasmid pRL-SV40 Renilla luciferase reporter (20 ng) was used as internal control. Supernatant was removed after 24 h and the cells were subsequently treated with CAPE for 12 h. Cell extracts were prepared and analyzed for luciferase activity using Dual-luciferase reporter assay system. Luciferase activity was expressed as relative luciferase activity (RLA).

### Statistical analyses

The results are presented as the mean ± SD where applicable. Data were analyzed using GraphPad Prism 5.0 and the Student's *t*-test to determine the level of significance. Statistical difference was accepted at p < 0.05. (GraphPad Prism 5.0 was used to perform statistical analysis.)

## Results and discussion

### Expressions of BLyS, TACI, BCMA and BAFF-R in human breast cancer cell lines

**Figure 1:**
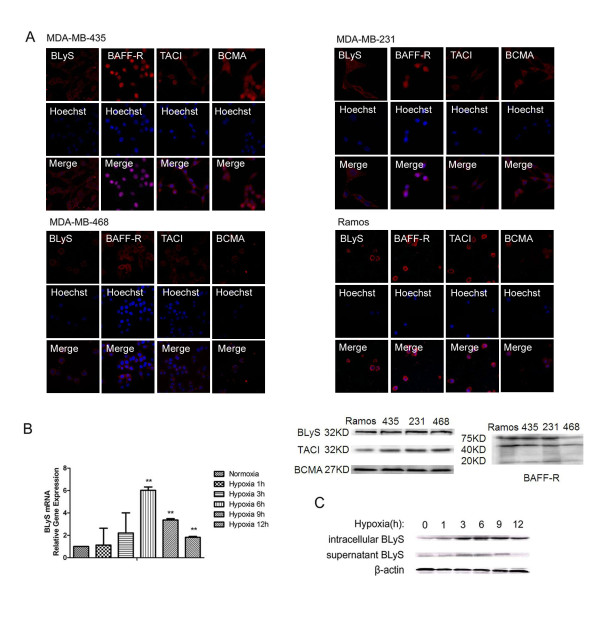
**Expressions of BLyS, TACI, BCMA and BAFF-R in human breast cancer cell lines**. (A) BLyS and its three receptors in human breast cancer cell lines MDA-MB-435, MDA-MB-231, MDA-MB-468 and B cell line Ramos by immunofluorescence (original magnification 200 ×) and Western Blotting. (B) The mRNA level of BLyS in the three cell lines were detected by real-time PCR under hypoxia for different time points. Data were means of triplicate samples with ± SD; vs normoxia, *, P < 0.05; **, P < 0.01; ***, P < 0.001. (C) BLyS protein level in MDA-MB-435 cells by Western Blotting analysis.

The expression of BLyS has been detected in monocytes, macrophages and T cells. The three receptors are mainly in B cells, T cells and several kinds of malignant cells [10]. It is reported that both BLyS and its receptors are present in Ramos cells [11,12]. As shown in Figure 1A, BLyS and the receptor proteins were present in MDA-MB-435, MDA-MB-231 and MDA-MB-468 cells by immunofluorescence and Western Blotting. Ramos cells were used as positive control. However, BAFF-R chiefly accumulated in the nucleus of MDA-MB-435 and MDA-MB-231 cells, indicating that BAFF-R may act as a transcription regulator of certain target genes including BLyS, CD154 and so on. It is reported that BAFF-R is capable of functioning both as a growth/survival cell membrane receptor, as well as a transcription factor or cofactor to promote B-cell survival and proliferation [13]. Further studies are necessary for confirming this hypothesis.

As shown in Figure 1B, the mRNA level of BLyS in MDA-MB-435 cell was dramatically increased in hypoxic conditions based on Q-PCR assay. In Figure 1C, protein level of BLyS was significantly elevated in hypoxic conditions for 3 h to 6 h. On the basis of Western Blotting data in MDA-MB-435 cells, we observed that BLyS was present not only as a dimer (~32 kDa) in plasma membrane and cytoplasm, but also as a trimer (~52 kDa) in supernatant. Both of the BLyS signals (~32 kDa and ~52 kDa) were strongly enhanced by the low oxygen tension.

### Migration of human breast cancer cells in the presence of BLyS

**Figure 2:**
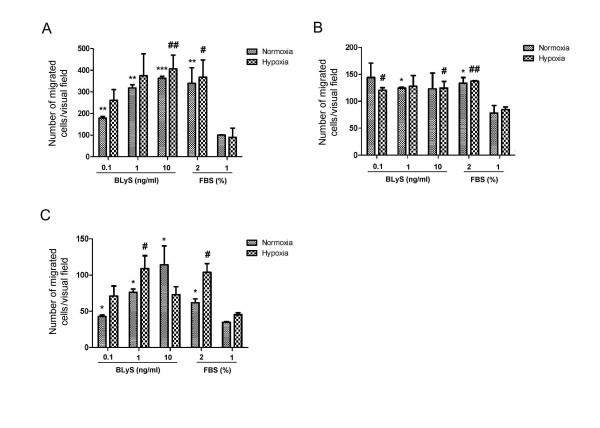
**Migration of human breast cancer cells in the presence of BLyS**. 0.1 ng/ml, 1 ng/ml and 10 ng/ml BLyS were added in the lower chamber. 2% FBS and 1% FBS added in the lower chamber were used as positive chemoattractant and negative chemoattractant respectively. (A) MDA-MB-435. (B) MDA-MB-231. (C) MDA-MB-468. Average numbers of cells migrating in five randomly selected fields were counted after 8 h (original magnification 200 ×). Data are means of triplicate samples with ± SD; *, P < 0.05, **, P < 0.01, ***, P < 0.001, vs 1% FBS under normoxia. #, P < 0.05, ##, P < 0.01, ###, P < 0.001, vs 1% FBS under hypoxia.

We determine breast cancer cells migration when treated with BLyS in both normoxic and hypoxic conditions. As seen in Figure 2, BLyS significantly enhanced the migration of MDA-MB-435, MDA-MB-231 and MDA-MB-468 cells in vitro compared with the negative control. The responses of the three cell lines to BLyS were different. BLyS treatment caused dose-dependent response in MDA-MB-435 and MDA-MB-468. However, no difference was found between the migration of MDA-MB-231 when treated with 10 ng/ml of BLyS compared to 0.1 ng/ml or 1 ng/ml of BLyS.

### Role of p65 activation in BLyS up-regulation

NF-kappa B is critical for the regulation of apoptosis, viral replication, tumorigenesis, inflammation and various autoimmune diseases. It is activated by a variety of stimuli such as hypoxia [14]. We also explored the possible involvement of HIF-1α which can be modulated by low oxygen tension in cells and tissues. HIF-1α leads to the transcriptional induction of a series of genes that participates in angiogenesis, iron metabolism, and glucose metabolism [15].

**Figure 3:**
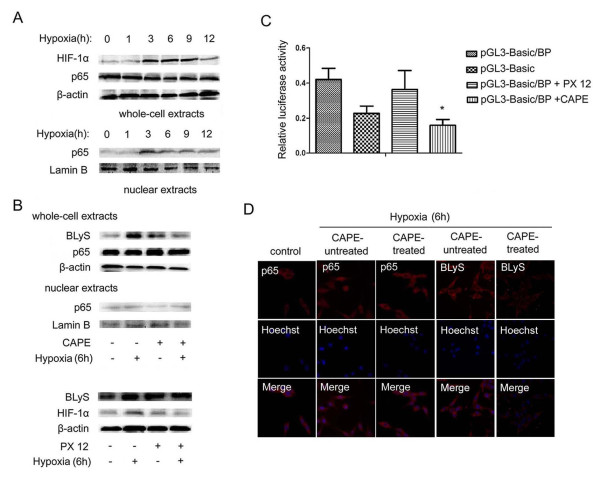
**Role of p65 activation in BLyS up-regulation**. (**A**) HIF-1α and p65 protein levels in MDA-MB-435 in hypoxic conditions for different time points by Western Blotting. (**B**) CAPE(50 μM)and PX 12 (10 μM) were used to determine the roles of p65 and HIF-1α in the regulation of BLyS expression by Western Blotting. The cells were treated with or without inhibitor in normoxic or hypoxic conditions for 6 h. (**C**) Effects of CAPE(50 μM)and PX 12 (10 μM) on BLyS promoter activity. Data were average luciferase activities of three independent transfections with ± SD. *, P < 0.05, vs pGL3-Basic/BP. (**D**) Localization of p65 protein and expression level of BLyS by immunofluorescence. MDA-MB-435 cells were challenged with CAPE (50 μM) for 6 h (original magnification 200 ×).

HIF-1α was up-regulated and p65 was translocated by hypoxia (Figure 3A). CAPE, a NF-kappa B antagonist, specifically inhibits NF-kappa B activation and PX 12 attenuates expressions of HIF-1α and VEGF. Decreased activation of p65 resulted in BLyS downregulation in MDA-MB-435 cells (Figure 3B). MDA-MB-435 cells were transfected with pGL3-Basic/BP plasmid and then treated with CAPE or PX 12 for 12 h. The RLA data suggested that CAPE rather than PX-12 decreased the BLyS promoter activity significantly (Figure 3C). Immunofluorescence showed that p65 could be activated by hypoxia and CAPE was against the activation. It also showed that CAPE blocked expression of BLyS in hypoxic conditions (Figure 3D). The preceding results showed that translocation of p65, rather than accumulation of HIF-1α, was responsible for BLyS up-regulation.

### Activation of akt protein involved in BLyS-enhanced cell migration

**Figure 4:**
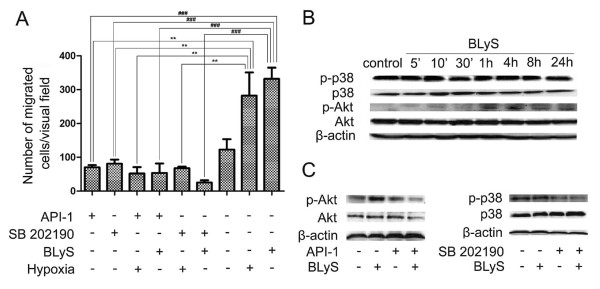
**Activation of Akt protein involved in BLyS-enhanced cell migration**. (**A**) Decreased number of migrated MDA-MB-435 cells was examined when the cells were treated with API-1 (10 μM) and SB 202190 (5 μM) for 8 h (original magnification 200 ×). Data were means of triplicate samples with ± SD; vs 2% FBS, **, P < 0.01; vs BLyS (10 ng/ml), ###, P < 0.001. (**B**) Phosphorylation of Akt and p38 MAPK proteins in MDA-MB-435 cells by Western Blotting analysis. (**C**) MDA-MB-435 cells were challenged with API-1 and SB 202190 for 4 h. API-1 inhibited the BLyS-induced (10 ng/ml) phosphorylation of Akt.

We have found that BLyS stimulated human breast cancer cell migration. Activation of Akt and p38 MAPK pathways might contribute to BLyS-enhanced cell migration. SB 202190 is a p38 MAPK antagonist and API-1 is an Akt/protein kinase B (PKB) antagonist. Enhanced migration of MDA-MB-435 cells in response to BLyS or 2% FBS was blocked by SB 202190 and/or API-1 (Figure 4A). MDA-MB-435 cells were treated with BLyS for 4 h, which led to the maximal phosphorylation levels of Akt protein (Figure 4B). As shown in Figure 4C, BLyS-induced phosphorylations of Akt could be abrogated by the specific inhibitors. But phosphorylation level of p38 MAPK induced by BLyS did not increase significantly as compared to the control. It suggested that inhibition by SB 202190 could be through another mechanism and BLyS-independent. In short, BLyS probably promoted breast cancer cell migration via Akt pathways.

## Discussion

We initially demonstrated that hypoxia modulated the expressions of BLyS and its receptors in human breast cancer cell lines. Our data also indicated enhanced breast cancer cell migration in response to BLyS in vitro. BlyS, an immunopotentiator, might be a potential therapeutic target in breast cancer treatment base on this study, but care should be taken for using immunopotentiator in cancer treatment. Cancer tissues consist of large amounts of mesenchymal cells including fibroblasts, endothelial cells, adipocytes as well as inflammatory cells. As we know, inflammatory cells are a major source of BLyS, suggesting that BLyS may act as a connection between inflammatory cells and cancer cells. Furthermore, growing evidences show that cancer can evolve from chronic inflammation [16]. Inflammation often accompanies cancer and recruits inflammatory cells which release plenty of inflammatory factors [17]. In addition, cancer-associated fibroblasts mediate cancer-enhancing inflammation [18]. Despite the relationship between inflammation and cancer is still poorly understood, it is believed that inflammatory cells are not the "street sweeper" in cancer tissues all along, but may trigger cancer progression [19]. Many other processes, such as EMT, are involved in the transition from inflammation to cancer [20]. It is prospected that an advanced breast cancer treatment could be developed if this field is much deeply explored.

Previous study reported that NF-kappa B played a key role in the transition from inflammation to cancer [21]. Cancer with NF-kappa B activity usually shows increased resistance to chemotherapy [22]. Furthermore, NF-kappa B is required for the expressions of many inflammatory genes [23]. Curcumin inhibited BLyS expression by decreasing the nuclear translocation of p65 in B lymphocyte cell lines [10]. Regarding HIF-1α, its protein level is extremely low in normoxic conditions. HIF-1α protein accumulates under hypoxia and regulates the target genes [8]. Interestingly, NF-kappa B also activates angiogenesis encoding genes HIF-1α and VEGF [24,25]. Mobility of cancer cells and cytokines productions are altered by hypoxia. All of these alterations will finally lead to angiogenesis, matrix degradation and metastasis in cancer. Cancer cells adapt to hypoxia for survival [26].

It is reported that BLyS suppresses the progression of several kinds of tumors and plays an important role in the development of immune system diseases [27]. However, our results showed an enhanced migratory in response to BLyS. Several reports support the critical roles of Akt and p38 MAPK in cancer cell survival, migration, apoptosis and anti-apoptosis [28,29]. Previous research indicated that BLyS led to rapid phosphorylations of Akt in B cells [30]. Our studies suggested that phosphorylations of Akt were essential for BLyS-enhanced cell migration in vitro.

## Conclusion

In conclusion, the results found that BLyS caused the enhanced migration of human breast cancer cells, while BLyS was up-regulated by hypoxia. However, further studies are required to confirm the mechanisms of BLyS action and reveal the relationship between inflammation and breast cancer progression.

## Competing interests

The authors declare that they have no competing interests.

## Authors' contributions

JZ proposed the study and wrote the first draft. LS and SSL modified the draft. RPZ contributed to the design of the study. LQZ and DDF helped analyzed the data. LC, JL and WTS aided with manuscript preparation. LYZ and STY provided the necessary funding. All authors read and approved the final manuscript.
